# Survey of transfemoral amputee experience and priorities for the user-centered design of powered robotic transfemoral prostheses

**DOI:** 10.1186/s12984-021-00944-x

**Published:** 2021-12-04

**Authors:** Chiara Fanciullacci, Zach McKinney, Vito Monaco, Giovanni Milandri, Angelo Davalli, Rinaldo Sacchetti, Matteo Laffranchi, Lorenzo De Michieli, Andrea Baldoni, Alberto Mazzoni, Linda Paternò, Elisa Rosini, Luigi Reale, Fabio Trecate, Simona Crea, Nicola Vitiello, Emanuele Gruppioni

**Affiliations:** 1 The BioRobotics Institute, Scuola Superiore Sant’Anna (Pisa), Viale Rinaldo Piaggio 34, 56025 Pontedera, Pisa Italy; 2 Dept. of Excellence in Robotics and AI, Scuola Superiore Sant’Anna (Pisa), Piazza Martiri della Libertà, 33, 56127 Pisa, Italy; 3 Centro Protesi INAIL – REPAIR Lab, Via Rabuina, 14, 40054 Vigorso di Budrio, Bologna Italy; 4 Healthcare Area, Fondazione ISTUD, Via Paolo Lomazzo, 19, 20154 Milano, Milan Italy; 5 Institute of Recovery and Care of Scientific Character (IRCCS), Fondazione Don Carlo Gnocchi Florence, Florence, Firenze Italy; 6 Dept. of Physical Medicine and Functional Re-Education, Istituto Palazzolo, Fondazione Don Carlo Gnocchi, Via Don Luigi Palazzolo, 21, 20149 Milano, Milan Italy; 7 Rehab Technologies, Istituto Italiano di Tecnologia (IIT), via Morego, 30, 16163 Genoa, Genoa Italy

**Keywords:** Transfemoral amputation, Lower-limb prostheses, Powered prosthesis, User-centered design, Human factors, Rehabilitation

## Abstract

**Background:**

Transfemoral amputees experience a complex host of physical, psychological, and social challenges, compounded by the functional limitations of current transfemoral prostheses. However, the specific relationships between human factors and prosthesis design and performance characteristics have not yet been adequately investigated. The present study aims to address this knowledge gap.

**Methods:**

A comprehensive single-cohort survey of 114 unilateral transfemoral amputees addressed a broad range of demographic and clinical characteristics, functional autonomy, satisfaction and attitudes towards their current prostheses, and design priorities for an ideal transfemoral prosthesis, including the possibility of active assistance from a robotic knee unit. The survey was custom-developed based on several standard questionnaires used to assess motor abilities and autonomy in activities of daily living, prosthesis satisfaction, and quality of life in lower-limb amputees. Survey data were analyzed to compare the experience (including autonomy and satisfaction) and design priorities of users of transfemoral prostheses with versus without microprocessor-controlled knee units (MPKs and NMPKs, respectively), with a subsequent analyses of cross-category correlation, principal component analysis (PCA), cost-sensitivity segmentation, and unsupervised K-means clustering applied within the most cost-sensitive participants, to identify functional groupings of users with respect to their design priorities.

**Results:**

The cohort featured predominantly younger (< 50 years) traumatic male amputees with respect to the general transfemoral amputee population, with pronounced differences in age distribution and amputation etiology (traumatic vs. non-traumatic) between MPK and NMPK groups. These differences were further reflected in user experience, with MPK users reporting significantly greater overall functional autonomy, satisfaction, and sense of prosthesis ownership than those with NMPKs, in conjunction with a decreased incidence of instability and falls. Across all participants, the leading functional priorities for an ideal transfemoral prosthesis were overall stability, adaptability to variable walking velocity, and lifestyle-related functionality, while the highest-prioritized general characteristics were reliability, comfort, and weight, with highly variable prioritization of cost according to reimbursement status. PCA and user clustering analyses revealed the possibility for functionally relevant groupings of prosthesis features and users, based on their differential prioritization of these features—with implications towards prosthesis design tradeoffs.

**Conclusions:**

This study’s findings support the understanding that when appropriately prescribed according to patient characteristics and needs in the context of a proactive rehabilitation program, advanced transfemoral prostheses promote patient mobility, autonomy, and overall health. Survey data indicate overall stability, modularity, and versatility as key design priorities for the continued development of transfemoral prosthesis technology. Finally, observed associations between prosthesis type, user experience, and attitudes concerning prosthesis ownership suggest both that prosthesis characteristics influence device acceptance and functional outcomes, and that psychosocial factors should be specifically and proactively addressed during the rehabilitation process.

**Supplementary Information:**

The online version contains supplementary material available at 10.1186/s12984-021-00944-x.

## Background

Limb loss imposes numerous physical, psychological, and social challenges on the individual, with a heavy impact on global health and quality of life, thus demanding the development of new strategies for managing daily life and dealing with changes in social relationships [1]. Among the approximately 40 million individuals with limb amputations worldwide [2], about 36 million (90%) are lower-limb amputees [3], of whom an estimated 26% are transfemoral amputees (TFAs) [4], for whom this host of challenges is compounded by the lack of a natural knee joint relative to trans-tibial (below-knee) amputees.

In this context, the prosthesis plays an essential role both in re-establishing functional autonomy and in substituting the lost limb as part of the individual’s body schema. While advances in prosthetic technology over the past decades have greatly increased the availability of powered prosthetic ankles [5, 6] and microprocessor-controlled knee units [7–9] for lower-limb amputees, there is presently just one commercially available knee unit [10] providing *positive power* (as opposed to merely modulating the joint impedance, i.e. stiffness), the adoption of which remains low due to the balance between weight, cost, usability, and functional benefit that it offers for most TFAs. Accordingly, TFAs continue to experience a host of mobility impairments and diminished quality of life [11, 12], while achieving lower satisfaction with their prostheses than trans-tibial amputees [13]. In particular, TFAs as a group exhibit significantly reduced gait speed, symmetry, and energetic efficiency, while experiencing greater postural instability and risk of falls [14–18]. The ability to maintain stable gait becomes especially impaired on inclined and uneven terrain [19]. While the use of prostheses with microprocessor-controlled knee units (MPKs) has been found to improve overall measures of gait stability and efficiency, functional balance, fall risk, and satisfaction [17, 20–22], conventional MPKs have difficulty recognizing and adapting to different gait speeds, inclines, stairs and other irregular terrain encountered in the community environment, which remain a formidable challenge for TFAs.

Despite the well-recognized importance of psychosocial outcomes such as functional independence (autonomy) and overall quality of life [23], a majority of research and development efforts on advanced transfemoral prostheses (TFPs) to date has focused on improving specific technical parameters such as velocity, torque, or power output [3, 24], on clinical measures such as gait quality and functional balance (tied to risk of falls) [20, 21], or on the user’s performance of functional tasks with the prosthesis [25–27]. While several studies have investigated user perceptions specific to single prosthesis characteristics such as cosmetic design [28] or the donning and doffing procedure [29], the precise relationships between TFP design features, functionality, and psychosocial outcomes remain insufficiently understood—and thus insufficiently accounted for in the TFP design process.

In this vein, Beckerle and colleagues [30] have proposed a detailed human-centered approach to lower-limb prosthesis design, with specific attention to the case of TFPs, emphasizing the need for equal consideration of human and technical factors. Moreover, a recent review has consolidated current knowledge on user priorities, design requirements, and clinical guidelines for upper-limb prosthesis selection and prescription, highlighting a widespread prioritization of comfort, natural appearance, and functional aspects such as the performance of activities of daily living, (ADL—especially eating and dressing) and the desire for sensory feedback [31]. While these findings likely reflect several universal prosthesis user needs such as comfort and functionality in ADLs, they cannot be globally generalized to lower-limb prostheses, given the differing roles of the upper and lower limbs in various ADLs and social interactions. Meanwhile, such a comprehensive synthesis has not been achieved specifically for TFPs.

The goal of the present study was to address this knowledge gap by illuminating the interplay between human and technical factors for TFP users, via a comprehensive survey of TFP user experience, functional capabilities, and design priorities. In particular, the study aimed (i) to characterize TFP users and their subjective experience of current commercial TFPs, and (ii) to identify user design priorities with respect to new generations of active robotic TFPs that may offer greater functionality, usability, and overall user appeal relative to current commercial prostheses.

## Methods

This study was based on an extensive survey of unilateral transfemoral amputees with the objective to characterize their subjective experience with their primary prostheses and their priorities regarding the features and functions of an ideal prosthesis, including a positively powered robotic knee unit. The survey format was chosen to achieve as large a sample as possible, via both in-person administration and online distribution throughout the affiliated clinical networks and patient groups. The questionnaire was custom-designed in collaboration between the Centro Protesi INAIL (Vigorso di Budrio, Italy), Scuola Superiore Sant’Anna (Pisa, Italy), the ISTUD Healthcare Area Research (Milan, Italy) and the Istituto Italiano di Tecnologia (Genoa, Italy). All study procedures, including subject recruitment and written informed consent were pre-approved by the designated ethics committee for the study sites, in conformance with all pertinent institutional protocols and the Declaration of Helsinki.

### Questionnaire

This study’s custom questionnaire (Table 1) was developed based on standard questionnaires assessing motor ability, function in activities of daily living (ADLs), prosthesis satisfaction, and quality of life in amputees, including the Trinity Amputation and Prosthesis Experience Scales-Revised (TAPES) [32], Questionnaire for Persons with a Transfemoral Amputation (Q-TFA) [33], Orthotics and Prosthetics Users’ Survey (OPUS) [34, 35], and Prosthesis Evaluation Questionnaire (PEQ) [36]. The questionnaire was administered in Italian (native language for all subjects) at a single time point (no follow-up) in one of two ways: i) via in-person interview by a physical therapist, at the Centro Protesi INAIL (Budrio, Italy) and the IRCCS Fondazione Don Gnocchi (Milan, Italy), or ii) via online self-administration.

**Table 1:** Summary of transfemoral prosthesis (TFP) user questionnaire

Category	Description	Question items	Question type & No
Section I. Retrospective evaluation—user characteristics and experience with current prosthesis			
Participant description	Clinical and demographic characteristics, and general description of the amputation and causes of the limb loss	Demographic Characteristics: Age, gender, region of residence, income, level of education obtained Amputation & Clinical Characteristics: year, anatomical level, side, etiology (traumatic vs. illness), condition of intact limb, time from amputation to first prosthesis outfitting Frequency of prosthesis use	8 multiple-choice; 4 free response
Current prosthesis description	Design features & details of the current prosthesis, including the socket, knee and foot	Prosthetic knee description: model, type (electronic vs. modular vs. skeletal) Prosthetic foot description (model, type) Socket support system (ischial seat vs. no ischial seat) Socket structure (rigid vs. semi-flexible) Use of socket liner (Y/N)	6 free response
Selection and satisfaction with current prosthesis	Subjective experience with the current prosthesis	Patient involvement in prosthesis selection Satisfaction with Prosthesis Function in ADL (Sat-Fn; 12 items)—gait on even and uneven ground, stairs (up, down), inclines (up, down; steep, gradual), sit-to-stand and stand-to-sit transitions, getting in & out of car, negotiating tight spaces Satisfaction with Usage & Maintenance Characteristics (6 items)—durability, reliability, cleanability, water resistance, battery life, charging time Satisfaction w. Comfort (3 items)—donning & doffing procedure, weight, noisiness Satisfaction w. Aesthetic Aspects (2 items)—general appearance, dimensions relative to body	Likert scale (1–6) 1 = low involvement/ satisfaction 6 = high involvement/ satisfaction
Prosthesis usage in daily life	Perceived autonomy in various ADL, as well as characterization of personal activities and prosthesis usage at home, at work, and during free time	Autonomy in ADL: stair ascent, stair descent (both step-over-step), gait on incline & decline (combined), sit-stand transitions (both directions, combined), bathing, dressing, housework, driving a car, managing & observing schedules, managing free time, attending public places Current activity at home /work/free time—free response Desired activity at home/work/free time—free response Time of prosthesis use at home/work/free time (Likert 1–6)	11 Likert scale (1–5): 1 = no autonomy (fully dependent) 5 = complete autonomy 7 multiple-choice 6 free response
Risk of falls	Incidence of falls in the last year and the principal causes of instability	Incidence of Falls: No. of falls in past year, main cause of fall Perceived Causes of Instability: stair ascent; stair descent; gait on incline (gradual; steep), gait on decline (gradual; steep), sit-to-stand; stand-to-sit; “other (describe)”	2 free response 11 binary choice
Pain	Phantom limb pain Joint pain in residual limb	Pain frequency (never; a few days a month; a few days a week; daily; always) Pain intensity (mild; moderate; severe; very severe; intolerable)	4 multiple choice
Socket	Skin problems; socket wear-and-tear and modifications needed over time	Skin problems Socket modifications (frequency, purpose)	2 free response
Subjective acceptance of prosthesis	Patient descriptions of their feelings about their prostheses	“What is the prosthesis for you?”	1 free response
Section II. Prospective evaluation—user priorities for an ideal prosthesis			
General characteristics of the ideal prosthesis	Priority of characteristics for an ideal transfemoral prosthesis	Comfort; Reliability; Cost; Weight; Battery life; Water resistance; Aesthetic aspects; Noisiness; Cleanability; Transportability	10-item rank-order scale
Functional characteristics of the ideal prosthesis	Priority of mobility-related functionality for an ideal transfemoral prosthesis	Stability, Functionality re: lifestyle, Adaptability to walking speed, Working activity functionality, Walking on uneven ground, Stair ascending, Functioning speed, Stair descending, Ramp walking, Running	10-item rank-order scale
Active assistance the prosthesis	“In which functions would you most prefer active assistance from your prosthesis?”	Moments of instability/balance loss; Stair ascent; Stair descent; Natural speed walking; fast walking; slow walking; Ramp ascent; Ramp descent; Standing up and sitting down	10-item rank-order scale
Adaptive socket	Preferred features & charact-eristics of the ideal socket	Breathable materials; Shape/volume adaptability; Variable rigidity; Cooling system; Topical drug release	5-item rank-order scale

Full survey available upon from authors upon request

Summarized in Table 1, the questionnaire consisted of 70 items, addressing four main thematic areas: user characteristics (clinical and demographic), perceived functional abilities and independence with the current prosthesis, user satisfaction and psychosocial experience, and priorities for an ideal prosthesis. User priorities for an ideal prosthesis were divided into four sub-categories, namely the general characteristics (Pr-GC), the limb interface socket (Pr-S), the mobility-related functionality(Pr-Fn), and the activities that would benefit from receiving active (i.e. positively powered) assistance (Pr-AA).Across all sections, survey items comprised a mix of question types, including multiple choice, Likert scale (1–6), rank ordering (1–10), and free written response.

### Study population: eligibility and inclusion

Subject eligibility was defined to include adult (age 18–79) unilateral TFAs, recruited from the patient populations at the participating rehab centers, as well as via online recruitment through their affiliated patient support networks on social media. Of surveys completed, selective response omissions were allowed, according to the user’s judgement of applicability to their case, while surveys with large blocks or whole sections of missing responses were omitted.

### Statistical analyses

Numeric survey data were summarized using quantitative descriptive statistics appropriate to each data type (continuous, categorical, ordinal), with ordinal data reported as median (inter-quartile range, IQR). In the case of design priority ranking data, missing responses were imputed a value of 10 (lowest rank order) for the purposes of clustering analysis, based on the interpretation that unrated factors were deemed insufficiently important to significantly influence the user’s choice of prosthesis or experience.

Based on the study objective of informing the design of an advanced robotic TFP, participants were grouped for analysis according to primary prosthesis type: those with electronic (i.e. microprocessor-controlled) knee units (MPKs), and those with purely mechanical (i.e. non-microprocessor-controlled) knees (NMPKs), including hydraulic knees. The use of MPKs was selected as the key variable of interest because MPKs represent the current state of the art among widely adopted TFP technology and have demonstrated significant differences in functional performance relative to other prosthesis types [20]. To assess for statistically significant differences between MPK and NMPK users, the non-parametric Mann Whitney U-test (α = 0.05) was applied for all continuous and ordinal variables, with the magnitude of between-group differences quantified using the common language (CL) effect size (f) [37]. The CL effect size describes the probability that in a random pair of samples taken one-from-each-group, the sample from a given group would have a larger value than that taken from the other. Thus, f values closer to 1 and to 0 (respectively) describe a greater effect size, while f = 0.5 describes a null effect size [38]. Between-group similarity was assessed using the Pearson chi-squared test for binary categorical variables (gender, amputation etiology: traumatic vs. non-traumatic), and using the Kolmogorov–Smirnov test to compare response distributions for ordinal variables (employment status, subjective prosthesis acceptance).

To address observed and potential demographic differences between groups in terms of overall health and lifestyle, autonomy data analysis was further stratified into traumatic and non-traumatic amputation sub-groups, as this etiological distinction was considered to provide the most reliable proxy indicator of overall health among the available variables, and to co-vary with other potential confounds such as age. Spearman rank correlations analysis was conducted to evaluate the relationship between various aspects of user experience, including prosthesis utilization (hours/day), autonomy, and different domains of satisfaction (aesthetics, functionality, and general characteristics). Given the wide variance in prioritization of cost, this correlation analysis was extended as well to a post-hoc sub-analysis assessing Spearman correlations between cost sensitivity (i.e. prioritization), income tier, and prosthesis payer, coded in ordinal fashion corresponding to the level of financial assistance: entirely individual (0); shared—national healthcare service with individual contribution (1); national healthcare service alone (2); INAIL (3). All quantitative data analysis was conducted using Excel, SPSS, and MATLAB software*.*

Answers to the free response questions “for me, my prosthesis is…” and “what were the causes of your fall(s) [as applicable]?” were analyzed qualitatively using the thematic analysis technique described by Braun and Clarke [39], which provides a theoretically flexible approach to analyze qualitative data for key descriptive information concerning the guiding research questions. Using this technique, recurring terms and concepts were used to synthesize thematic categories related to user perceptions of their prostheses and their functional abilities, and each response was then semantically classified to one of the identified themes.

### Cost sensitivity analysis and user segmentation (clustering of priorities)

Given the real-world role of cost in constraining both the design and selection of TFPs, the variation in user priorities as a function of cost sensitivity was explored. To clarify the relationship between cost sensitivity and other user priorities, users across both NMPK and MPK groups were segmented into tiers based on their prioritization of cost: high (rank 1–3), medium (4–6), or low (7–10), and distributions of priorities were visually compared across Pr-GC, Pr-Fn, and Pr-AA categories. To further distinguish user priorities among the most cost-sensitive users, a post-hoc clustering analysis was performed via unsupervised K-Means clustering within the user segment rating cost among their top 3 Pr-GC priorities.

As a noise reduction measure to facilitate a cleaner segmentation of TFP users in each category, the dimensionality of raw survey data was first reduced using principal component analysis, via transformation into the minimum number of dimensions (d_85_) capturing at least 85% of total variance. PCA for rank order data was performed via MATLAB function *pcacov*, using Spearman rank order correlations, with a rank of 10 (‘not important') imputed to all missing data points. Data were then reverse-transformed back into the original n-dimensional space, and clustering was then performed on transformed data using a non-hierarchical K-Means clustering algorithm (executed in MATLAB) for K = 3 clusters, with 100 random initializations per instance (k) and iteration run until convergence. As indicated for the standard K-Means algorithm, the net cost function for each segmentation was computed as the root-mean-square (RMS) distance from each n-dimensional data point to its respective cluster centroid [40]. Finally, to identify specific priorities that differed significantly across the three cost sensitivity tiers, Kruskal–Wallis tests were applied across the tiers, individually for each priority in the Pr-GC, Pr-Fn, and Pr-AA categories. For priorities exhibiting significant overall differences, post-hoc. Mann-Whitney U tests were conducted to identify pair-wise significant differences between cost sensitivity tiers.

## Results

### Demographic and clinical characteristics

A total of 114 correctly completed questionnaires were obtained from unilateral TFA participants from the 189 surveys administered (based on available recruitment capacity) during the period of February-April, 2018, with 75 excluded for incompleteness or failure to meet inclusion criteria (e.g. trans-tibial amputees). Summarized in Table 2, the final subject sample comprised mostly males (77.2%), with a median (IQR) age of 50 (15) years and unilateral amputations occurring at a median (IQR) age of 30 (22.5) years, corresponding to median (IQR) 16.5 (26.3) years since amputation. Among all subjects, 26.3% of amputation cases were semi-acute (≤ 2 yr), 8.8% recent (2-5 yr), and 64.9% chronic (> 5 yr). Those using MPKs and NMPKs (respectively) as their primary prostheses were comparable in age and time between amputation and receipt of the first prosthesis, though statistically significant between-group differences were noted in amputation etiology (traumatic amputations were more prevalent), gender (males were more prevalent), age at the time of amputation (older for NMPK), and time since amputation (longer for MPK).

**Table 2:** Clinical demographics of study cohort, by group

Variable	Overall (N = 114)	NMPK (n = 45) n (%)**	MPK (n = 69)	p*
Amputation etiology				
Traumatic	90 (78.9%)	27 (60%)	63 (91.3%)	< **0.001**
Non-traumatic (NT)	24 (21.1%)	18 (40%)	6 (8.7%)	
NT—dysvascular (diabetic)	7 (6.1%)	6 (13.3%)	1 (1.4%)	
NT—cancer	8 (7.0%)	5 (11.1%)	3 (4.3%)	
NT—congenital deformities	3 (2.6%)	2 (4.4%)	1 (1.4%)	
NT—surgical complications & infections (secondary)	6 (5.3%)	4 (8.9%)	2 (4.4%)	
Gender				
Men	88 (77.2%)	30 (66.7%)	58 (84.1%)	**0.011**
Women	22 (19.3%)	14 (31.1%)	8 (11.6%)	

		Med (IQR)		
Age (*a*)	50 (15)	51 (22)	49.5 (13)	0.610
Age at amputation (*a*)	30 (22.5)	39 (31.5)	29 (21)	**0.001**
Time since amputation (*a*)	16.5 (26.3)	4.5 (19)	19.5 (26.8)	** < 0.001**
Time from amputation to 1^st^ TFP (mo.s)	6 (5)	6 (5.25)	6 (4)	0.760

^*^Reported p-values for significant differences in distribution between NMPK and MPK user groups, using Pearson chi-squared test for categorial parameters (gender, etiology) and from Mann–Whitney U-test for all others. Significant differences highlighted in bold. **Subtotals less than 100% are due to individual missing responses

Table 3 Summarizes the data on subject income, occupation, employment status, impact of amputation on work ability. Overall, there were no statistically significant differences between MPK and NMPK users in any of these work or income-related categories, despite the observed greater percentages of MPK users in physical occupations prior to amputation (81.6%MPK vs. 65.7% NMPK, p = 0.08) and the lower percentage of MPK users performing the same occupation following amputation (38.1% NMPK vs. 23.1% MPK, p = 0.095). Accordingly, there was no significant between-group difference in the reported impact of amputation on vocational ability.

**Table 3:** Work and income

	Overall	NMPK	MPK	p*
Employment status				
Stable Employment	60	20	40	> 0.999
Part-time Employment	5	2	3	
In search of work	9	3	6	
Don't work	12	7	5	
Student	3	1	2	
Retired	22	11	11	
Homemaker	2	1	1	
Total	114	45	68	
Physical occupation (prior)?				
Y	72	23	49	0.0800
N	23	12	11	
Same occupation as before amputation?				
Y	31	16	15	0.0945
N	76	26	50	
Negative influence of amputation on work ability (1–4)	3(3)	3(3)	3(3)	0.9834

		Med (IQR)		
Annual income	1 (1)	1 (1)	1 (2)	
Decline to specify	32	6	26	0.6407
< 18,000€	43	21	22	
18,000–36,000 €	20	10	10	
36,000–70,000 €	12	6	6	
70,000–100,000 €	7	2	5	
> 100.000 €	0	0	0	

^*^P values computed using chi-squared test for binary questions, 2-sample Kolmogorov–Smirnov test for categorial variables, and Mann–Whitney U test for ordinal variables

For further reference regarding how additional prosthesis components (foot, socket, liner) may influence user experience or priorities, the main types and features of these components among participants are summarized in Table 4.

**Table 4:** Transfemoral prosthesis (TFP) characteristics, by group

Characteristic	Overall (N = 114)	NMPKs (n = 45; 40%)	MPKs (n = 69; 60%)
Prosthetic foot type			
SACH	8 (7%)	6 (13.3%)	2 (2.9%)
Articulated (Single or Multi-Axis)	15 (13.2%)	7 (15.6%)	8 (11.6%)
Carbon fiber	71 (62.3%)	22 (48.9%)	49 (71%)
*Missing/other*	20 (17.5%)	10 (22.2%)	10 (14.5%)
Socket support system			
Ischial support	68 (59.6%)	23 (51.1%)	45 (65.2%)
Ischial containment	35 (30.7%)	15 (33.3%)	20 (29%)
*Missing/other*	9 (9.7%)	7 (15.6%)	4 (5.7%)
Socket structure frame			
Entirely rigid	38 (33.3%)	19 (42.2%)	19 (27.5%)
Windows (semi flexible)	71 (62.3%)	21 (46.7%)	50 (72.5%)
*Missing/other*	5 (4.4%)	5 (11.1%)	0
Liner			
Yes	61 (53.5%)	22 (48.9%)	39 (56.5%)
No	48 (42.1%)	19 (42.2%)	29 (42%)
*Missing*	5 (4.4%)	4 (8.9%)	1 (1.4%)

### User experience & satisfaction

#### Functional capabilities & independence

*Independent Prosthesis Usage* (Table 5). Reported prosthesis utilization was high across all participants, with a median (IQR) usage frequency of 7(0) days per week in both groups, and with MPK users indicating significantly greater daily use of the prosthesis. This difference varied by context, with both groups reporting comparably high usage at work/school, but MPK users reporting greater usage at home and in recreational settings. MPK users also noted significantly greater involvement in the prosthesis selection process.

**Table 5:** Utilization of current prosthesis

Variable	Overall (N = 114)	NMPK (n = 45)	MPK (n = 69)	*p*^*c*^	*f*^*c*^
	Median (IQR)				
Involvement in prosthesis selection^a^	5 (3.5)	4 (3)	6 (1)	< 0.001	0.814
Prosthesis utilization					
Days per week	7 (0)	7 (0)	7 (0)	< 0.001	0.616
Hours per day	14 (6)	10 (7)	15 (2)	< 0.001	0.783
Frequency of Prosthesis Use, by context^b^					
At work	5 (0)	5 (2)	5 (0)	< 0.001	0.655
At home	5 (2)	3 (3.5)	5 (1)	< 0.001	0.783
Free time	5 (1)	4 (3)	5 (0)	< 0.001	0.717

^a^Values on Likert Scale from 1 (low) to 6 (high) involvement/satisfaction. ^b^Likert values of 1–5 describing frequency of use. ^c^All statistical comparisons performed via Mann–Whitney U Test (*p*), with common language effect size (*f*)

**Fig. 1:**
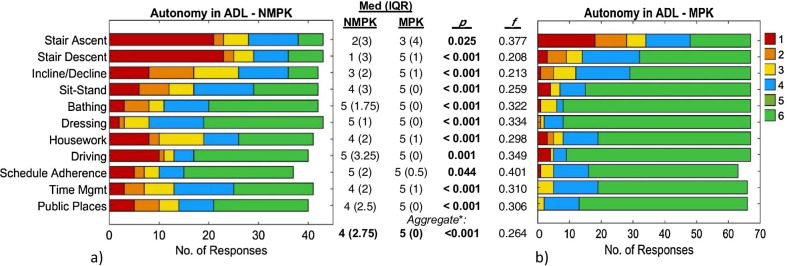
Autonomy in Activities of Daily Living (ADL): Distribution of Likert scale responses, ranging from “completely autonomous” (5) to “completely non-autonomous [fully dependent]” (1) for each task in a) MPK and b) NMPK users. Significance (p) and common language effect size (f) reported for Mann–Whitney U Test (α = 0.05). *: Aggregate autonomy computed as the median (IQR) of each subject’s median autonomy across all constituent ADL

*Autonomy.* Perceived autonomy for various ADLs is depicted in Fig. 1, with the consistent trend of MPK users rating themselves as more autonomous than NMPK users for all tasks. These differences were statistically significant for all tasks except for stair ascent, with the strongest effect sizes (f (f ~ 0.74–0.79) in the kinematically specific tasks of stair descent, gait on inclines/declines, and sit-stand transitions. By contrast, more general and varied ADLs such as housework, free time management, and negotiation of public spaces exhibited mild-to-moderate effect sizes in the range of f ~ 0.60–0.70. While a majority of MPK users rated themselves as fully autonomous in all tasks (median(IQR) 5(0)) except for stair ascent (median(IQR) 3(4)), NMPK autonomy ratings varied more widely by task, with predominantly *non-*autonomous ratings (median score ≤ 3 of 6) in the tasks of stair ascent, descent, and ramp walking. Notably, a majority of TFP users in both groups reported full functional autonomy in the tasks of dressing, bathing, driving a car, and schedule management.

Concerning amputation etiology, stratified analyses within the traumatic and non-traumatic amputee sub-groups found the differences between MPK and NMPK users to remain evident for a majority of ADLs (Table 6), though the set of tasks remaining significantly different varied somewhat by etiology. Tasks with significantly greater MPK autonomy in both etiologies included stair descent, incline/decline gait, sit-stand transitions, bathing, and attending public places. For all ADL, the MPK vs. NMPK effect sizes were notably stronger within the non-traumatic sub-group, with f values greater than 0.75 for all significant differences, as compared to the moderate effects (f ~ 0.6–0.75) among traumatic amputees.

**Table 6:** Autonomy in ADL—stratified by amputation etiology

*“Overall, to what extent do you feel independent in performing the following activities?”*								
Activity	NMPK_Tr_ (n = 26)	MPK_Tr_ (n = 61)	*p*^*c*^	*f*^*c*^	NMPK_NT_ (n = 17)	MPK_NT_ (n = 6)	*p*^*c*^	*f*^*c*^
	Median^a^ (IQR)				Median^a^ (IQR)			
Stair ascending	3 (3)	4 (4)	0.4769	0.547	1 (1.25)	2.5 (3)	0.1293	0.691
Stair descending	3 (3)	5 (1)	**0.0006**	**0.723**	1 (1.25)	5 (1)	**0.0007**	**0.941**
Ramp walking	4 (2)	5 (1)	**0.0007**	**0.719**	2 (2)	5 (1)	**0.0030**	**0.912**
Sit-to-stand/Stand-to-sit	4 (2)	5 (0)	**0.0022**	**0.674**	3 (2)	5 (0)	**0.0081**	**0.870**
Bathing	5 (1)	5 (0)	**0.0171**	**0.610**	3.5 (3)	5 (0)	**0.0431**	**0.776**
Dressing	5 (1)	5 (0)	0.0719	0.579	4 (2)	5 (0)	**0.0273**	**0.794**
Housework	4 (2)	5 (1)	**0.0192**	**0.637**	3 (3)	5 (2)	0.0734	0.750
Driving a car	5 (0)	5 (0)	0.4775	0.531	1 (2)	5 (0)	**0.0242**	**0.800**
Work/study schedule adherence	5 (0.5)	5 (0)	0.8230	0.512	3 (4)	4.5 (1)	0.1108	0.731
Management of free time	5 (2)	5 (0.5)	**0.0299**	**0.621**	3 (1.75)	4 (2)	0.2113	0.678
Attending public places	5 (1)	5 (0)	**0.0362**	**0.610**	3 (3)	5 (0)	**0.0112**	**0.857**
Aggregate^b^	5 (1)	5 (0)	**0.0156**	**0.628**	3 (2)	5 (1)	**0.0047**	**0.892**

^a^Values reported on Likert scale from 1 (fully dependent, not autonomous) to 5 (fully autonomous); Reported separately for traumatic (Tr) and non-traumatic (NT) amputees with and without microprocessor-controlled knees (MPKs), respectively

^b^Aggregate autonomy computed as the median (IQR) of each subject’s median autonomy across all constituent ADL

^c^All statistical comparisons conducted using Mann–Whitney U test, reported with common language effect size (f). Significant differences highlighted in bold

*Falls* 72% of the whole group (80% NMPK vs. 67% MPK) reported at least one fall within the past year, with stair descent and steep ramp descent cited as the most frequent situational causes of instability (Table 7). Steep ramp ascent and gradual slope descent were also cited as common causes of instability by both groups. While not reaching significance (p = 0.059), the greatest-magnitude *difference* in the attribution of instability between groups was for other (miscellaneous) causes, cited by more MPK (19%) than NMPK users (3%). Based on their attributions of specific recent falls in free responses, NMPK users most commonly cited the aforementioned environmental factors associated with instability, while MPK users commonly attributed falls to personal errors and controllable factors such as “distraction” (n = 6), “tripping” (n = 4), as well as (n = 1) attribution to “rapid transitional movements”.

**Table 7:** Falls and situational causes of instability

Variable	Overall (N = 114) (%)	NMPK (n = 45) (%)	MPK (n = 69) (%)	Chi^2^	*p*
Falls within the past year					
Yes	82 (72)	36 (80)	46 (67)	2.736	0.098
No	29 (25)	8 (18)	21 (30)		
*Missing*	3 (3)	1 (2)	2 (3)		
Situations of perceived of instability (‘*select all that apply*’)					
Stair ascent	8 (7)	5 (8)	3 (5)	1.909	0.167
Stair descent	26 (22)	14 (23)	12 (21)	2.912	0.088
Ramp ascent (steep)	16 (13)	10 (16)	6 (10)	**4.130**	**0.042**
Ramp descent (steep)	28 (24)	14 (23)	14 (24)	1.721	0.190
Slope ascent (gradual)	7 (6)	4 (7)	3 (5)	0.975	0.324
Slope descent (gradual)	12 (10)	7 (11)	5 (9)	1.997	0.158
Sitting down	4 (3)	2 (3)	2 (3)	0.192	0.661
Standing up	5 (4)	3 (5)	2 (3)	0.922	0.337
Other	13 (11)	2 (3)	11 (19)	3.564	0.059

Significant differences highlighted in bold

#### Prosthesis satisfaction & psychosocial experience

**Fig. 2:**
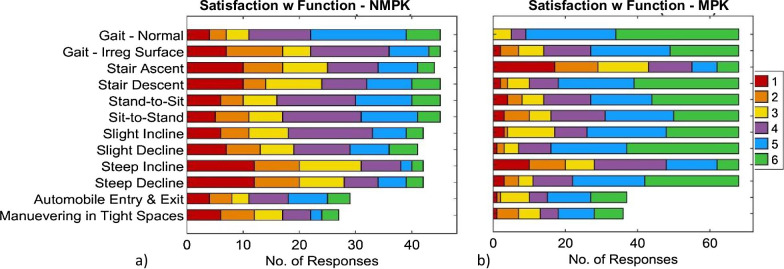
Satisfaction with Prosthesis Functionality in ADL in A) all MPK and B) all NMPK users (traumatic + non-traumatic). Likert scale responses range from “completely satisfied” (6) to “completely dissatisfied” (1). Corresponding summary data reported in Table 8

*Satisfaction* with the current prosthesis is summarized in Table 8 for the four categories of prosthesis functionality, comfort, aesthetics, and general characteristics & maintenance, with MPK users reporting significantly greater global satisfaction in all categories, as well as for a majority of specific ADL. Stair ascent stood out as the task with lowest prosthesis satisfaction (median (IQR): 3 (2)) among MPK users, exhibiting no significant difference relative to NMPK users. To provide a better idea of priority ranking distribution across groups, the full response distributions regarding ADL-specific prosthesis functionality are depicted in Fig. 2.

**Table 8:** Satisfaction with the current prosthesis

Variable	Overall	NMPK	MPK	*p*	*f*
	Median (IQR)^*^				
Satisfaction rating					
Comfort*	5.0 (2)	4.0 (1)	5.0 (1)	** < 0.001**	**0.696**
Donning and doffing	5.0 (2)	4.0 (2.25)	5.0 (1)	**0.008**	**0.642**
Weight	4.0 (2)	4.0 (2)	4.0 (1)	0.081	0.585
Noisiness	6.0 (1)	5.0 (2)	6.0 (1)	** < 0.001**	**0.678**
Aesthetic aspects*	5.0 (2)	4.0 (1.5)	5.0 (1.5)	** < 0.001**	**0.694**
General appearance	5.0 (2)	4.0 (2)	5.0 (2)	** < 0.001**	**0.684**
Size/dimensions relative to body	5.0 (2)	4.0 (2)	5.0 (2)	**0.002**	**0.679**
Functionality*	4.5 (1.5)	4.0 (2.25)	5.0 (1.5)	** < 0.001**	**0.792**
Gait	5.0 (2)	5.0 (1)	5.0 (1)	** < 0.001**	**0.752**
Gait on irregular surfaces	4.0 (2)	4.0 (2)	5.0 (2)	** < 0.001**	**0.747**
Stair ascent	3.0 (2)	3.0 (2)	3 (2.25)	0.675	0.528
Stair Descent	5.0 (3)	3.0 (3)	5.0 (2)	** < 0.001**	**0.771**
Stand-to-sit transitions	4.0 (3)	4.0 (2)	5.0 (2)	**0.004**	**0.665**
Sit-to-stand transitions	4.0 (2)	4.0 (2)	5.0 (2)	**0.015**	**0.642**
Ascent of gradual incline (ramp)	4.0 (2)	4.0 (1.75)	5.0 (2.25)	** < 0.001**	**0.713**
Descent of gradual decline (ramp)	5.0 (2)	4.0 (3)	5.0 (1)	** < 0.001**	**0.782**
Ascent of steep incline (ramp)	3.0 (2)	3.0 (2.75)	4.0 (3)	**0.001**	**0.667**
Descent of steep decline (ramp)	4.0 (3)	3.0 (3)	5.0 (2)	** < 0.001**	**0.806**
Getting in and out of automobile	4.5 (2)	4.0 (3)	5.0 (3)	0.906	0.638
Maneuvering in tight spaces	4.0 (3)	3.0 (2)	4.5 (2)	0.681	0.695
General Characteristics & Maintenance*	5.0 (2)	5.0 (1.0)	5.5 (1)	**0.003**	**0.665**
Cleanability	5.0 (2)	4.0 (2)	6.0 (1)	**0.002**	**0.671**
Robustness	5.0 (2)	5.0 (1.5)	5.0 (1)	**0.018**	**0.627**
Water Resistance	3.0 (4)	3.0 (4)	3.0 (4)	0.353	0.524
Reliability	5.0 (2)	5.0 (2)	5.0 (1)	**0.012**	**0.617**
Battery autonomy	5.0 (2)	N/A	5.0 (2)	N/A	N/A
Battery charging time	6.0 (1)	N/A	6.0 (1)	N/A	N/A

^*^Aggregate median (IQR) values for each category computed as the median and IQR of each subject’s median satisfaction across constituent questions. Statistical significance (p) computed using Mann–Whitney U test, with common language effect size (f). Significant differences highlighted in bold

Table 9 reports the Spearman correlations (and corresponding p values) between daily prosthesis usage (hours/day), overall (aggregate) autonomy, and aggregate satisfaction ratings for, the comfort, aesthetics, functionality, and general characteristics sub-categories. While all between-category correlations were significant, those with the greatest (albeit only moderate) magnitude (rho ~ 0.5) were noted between autonomy and satisfaction with both prosthesis function (rho = 0.434) and general characteristics (0.556), respectively. Comparably strong correlations were also noted between all respective sub-categories of prosthesis satisfaction. While hours of daily prosthesis use exhibited significant positive correlations with median aggregate autonomy (rho = 0.277, p = 0.004) and all categories of prosthesis satisfaction, the magnitudes of these correlations were comparatively weak.

**Table 9:** Rank correlations between daily usage (hours/day), aggregate autonomy, and aggregate prosthesis satisfaction (by category)

	Daily usage	Auton	Sat-comfort	Sat-aesth.	Sat-function	Sat-Gen char
	*rho*					
Daily use	1.00	0.277	0.221	0.208	0.258	0.154
Auton	0.277	1.00	0.234	0.362	0.434	0.556
Comfort	0.221	0.234	1.00	0.491	0.489	0.503
Aesthetics	0.208	0.362	0.491	1.00	0.455	0.491
Function	0.258	0.434	0.489	0.455	1.00	0.465
Gen Char	0.154	0.556	0.503	0.491	0.465	1.00

	*p*					
Daily use	1.00	0.004	0.025	0.036	0.009	0.123
Auton	0.004	1.00	0.018	0.0002	< 0.0001	< 0.0001
Comfort	0.025	0.0178	1.00	< 0.0001	< 0.0001	< 0.0001
Aesthetics	0.036	0.0002	< 0.0001	1.00	< 0.0001	< 0.0001
Function	0.009	< 0.0001	< 0.0001	< 0.0001	1.00	< 0.0001
Gen Char	0.1232	< 0.0001	< 0.0001	< 0.0001	< 0.0001	1.00

Spearman rank correlations and p values for categories of daily prosthesis usage (hours/day), plus median autonomy (Auton.), and satisfaction with comfort, aesthetic aspects (Aesth.), mobility-related functionality (function), and general characteristics (Gen Char)

*Subjective Prosthesis Acceptance & Body Integration* Thematic analysis of free responses to the question “*what is the prosthesis for you?*” revealed 3 semantic categories: “tool”, describing the TFP as a means to obtain a functional result; “part of me”, describing it as a naturalistic part of the body; and “obstacle/noise”, as an impediment to daily life. Across all subjects, approximately half (51% overall; 49% MPK; 53% NMPK) described the TFP as a “tool”, 38% as “part of me”, and only 6% as an obstacle. To evaluate between-group differences in subjective acceptance and body integration, an ad-hoc Kolmogorov–Smirnov analysis was applied by interpreting responses as an ordinal variable representing the distribution of the sentiment of prosthesis acceptance/integration across these 3 semantic categories (“obstacle” = low; “tool” = medium; “part of me” = high). This analysis did not reveal a significant overall between-group difference in subjective prosthesis integration (p = 0.320), despite the notable differences in proportions of MPK versus NMPK users (46.2% vs. 30.2%) characterizing their prostheses as “part of me” and as obstacle/noise (1.5% MPK vs. 14.0% NMPK) – likely due to similar portions describing their prostheses as a tool (52.3% NMPK vs. 55.8% NMPK). 4% of MPK and 5% of NMPK subjects (respectively) did not complete this question item.

### Design priorities for an ideal transfemoral prosthesis (TFP)

**Fig. 3:**
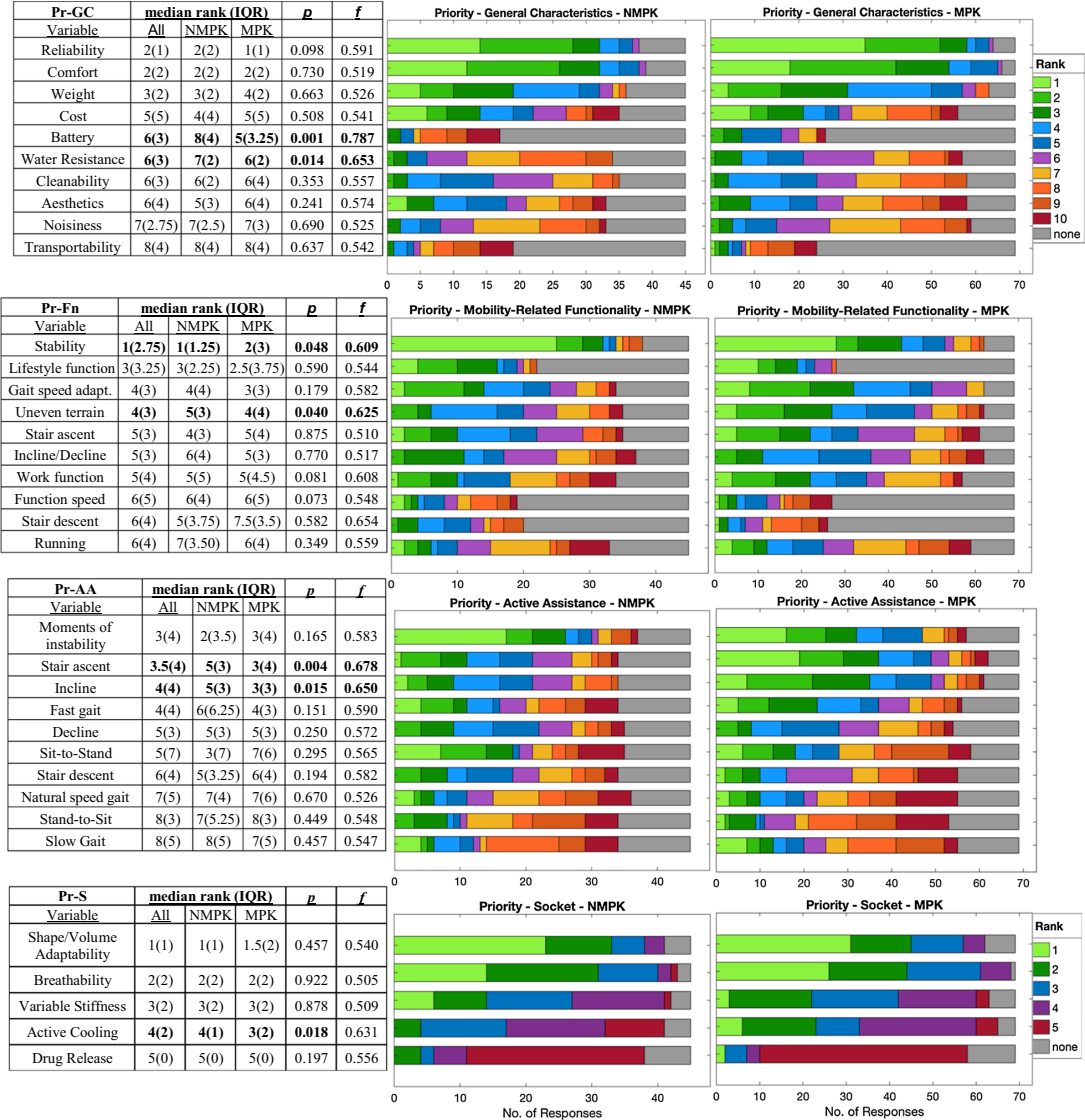
Design Priorities for an ideal transfemoral prosthesis (TFP) for the categories of general characteristics (Pr-GC), mobility-related functionality (Pr-Fn*), active assistance (Pr-AA), and socket (Pr-S)). Significance (p) assessed using Mann–Whitney U tests, with common language effect size (f) on all valid response data, omitting missing responses. *: An initial survey version without the questions of lifestyle functionality, functioning speed, or stair descent was issued was issued to a total of 50 participants (15 NMPK, 35 MPK), accounting for the outsized number of missing responses to these items

User priority rankings for the features and functions of an ideal TFP are summarized in Fig. 3 for all four design categories evaluated by the questionnaire. For general prosthesis characteristics (Pr-GC), reliability, comfort, and prosthesis weight scored as the highest-ranked items for both the NMPK and MPK groups, with cost ranking fourth. Slight yet significant differences in priorities between groups across all categories included the higher prioritization of overall stability by NMPK users (p = 0.048, f = 0.609), while MPK users gave significantly higher priority to gait on uneven terrain (p = 0.040, f = 0.625), battery life (p = 0.001, f = 0.787), water resistance (p = 0.014, f = 0.653), active cooling (p = 0.018, f = 0.509) and active assistance while ascending stairs (p = 0.004, f = 0.678) and inclines (p = 0.015; f = 0.650).

Regarding functional mobility (Pr-Fn), overall prosthesis stability was the highest-rated priority for the whole sample (including a majority of NMPK users ranking it in the first position), with lifestyle-related functionality ranking second (Fig. 3). Gait speed adaptability ranked as the third-highest priority for both groups, while MPK users expressed a higher preference towards walking on uneven terrain. Concerning contexts for active assistance from the prosthesis (Pr-AA), moments of instability were ranked highest by both groups, though no task earned an mRO of 1 due to high individual variability. Regarding the prosthetic socket (Pr-S), both groups prioritized (1) socket adaptability to the residuum shape and volume, (2) the use of breathable materials, and (3) variable socket stiffness, differing only regarding an active cooling system, ranked slightly higher by MPK users.

### Principal component analysis, user segmentation, and cost sensitivity sub-analysis

As a first step to understanding how TFP users might best be distinguished and grouped based on their design priorities, Principal Component Analysis conducted separately in the areas of Pr-GC, Pr-Fn, and Pr-AA yielded the primary principal components (PCs) summarized in Table 10. Overall, there was a moderately high degree of dimensional reduction possible in these categories, with the first four PCs capturing upward of 64% of total sample variance within each category (64.4% Pr-GC; 76.8% Pr-Fn; 68.0% Pr-AA). Moreover, the combinations of dominant variable weights within each PC, which represent salient positive and negative correlations between user priorities, were found to consist of functionally relevant groupings of variables that allow the interpretation of PCs as aggregate higher-order measures of user priorities (Table 10). A complete listing of PC loadings is provided in Additional file 1: Tables S1–S3.

**Table 10:** Summary of principal component decompositions of survey data

Category	PC ID	Component score (PVC^a^) (%)	Dominant positive correlation(s)^b^	Dominant negative correlation(s)^b^
Priority-Function	Pr-Fn-PC1	37.8%	Speed of Functioning, Lifestyle functionality; Stair Descent	Incline & Decline
Priority-Function	Pr-Fn-PC2	14.5%	Stair Ascent	Adaptability to velocity; function re: work
Priority-Function	Pr-Fn-PC3	13.4%	Stability of support (1°); adaptability to gait speed (2°)	Running
Priority-Function	Pr-Fn-PC4	11.1%	Work functionality (1°); Stair Ascent (2°)	Uneven terrain
Priority—Active Assistance	Pr-AA-PC1	22.4%	Sit-to-stand, stand-to-sit	Fast gait, stair descent, decline gait
Priority—Active Assistance	Pr-AA-PC2	18.4%	Gait on level ground; gait at usual speed	Decline gait, stair descent
Priority—Active Assistance	Pr-AA-PC3	16.1%	Gait incline, stair ascent	Moments of instability
Priority—Active Assistance	Pr-AA-PC4	11.1%	Instability recovery, Level gait at usual speed, incline	Fast gait, stair descent
Priority—Characteristics	Pr-GC-PC1	20.3%	Battery & Transportability	Aesthetics, noisiness
Priority—Characteristics	Pr-GC-PC2	17.4%	Water resistance, weight, reliability,	comfort
Priority—Characteristics	Pr-GC-PC3	14.8%	Cost	Cleanability
Priority—Characteristics	Pr-GC-PC4	11.9%	Weight & Portability	Reliability, noisiness

^a^Percentage of variance captured (PVC); ^b^Within each PC, dominant positive correlations are identified as the variables with positive variable weights greater than 0.3, while dominant negative correlations are the variables with *negatively signed* weights greater than 0.3 in magnitude. For complete list of constituent variable weights, see Additional file 1: Tables S1–S3

**Fig. 4:**
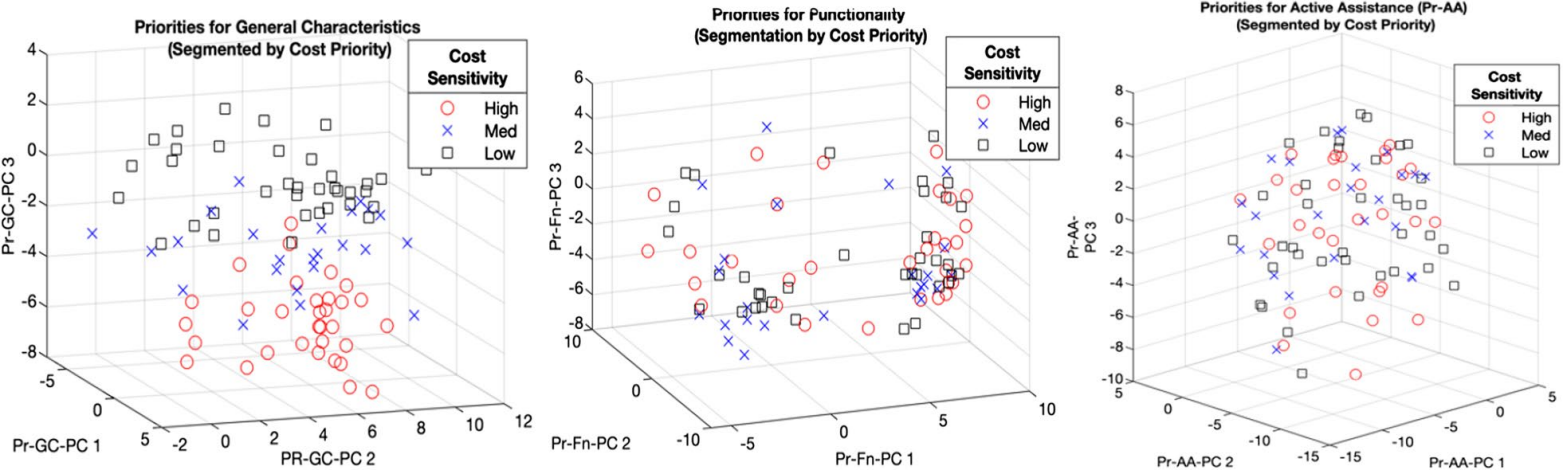
Distribution of user priorities in principal component (PC) space, segmented by cost sensitivity

**Fig. 5:**
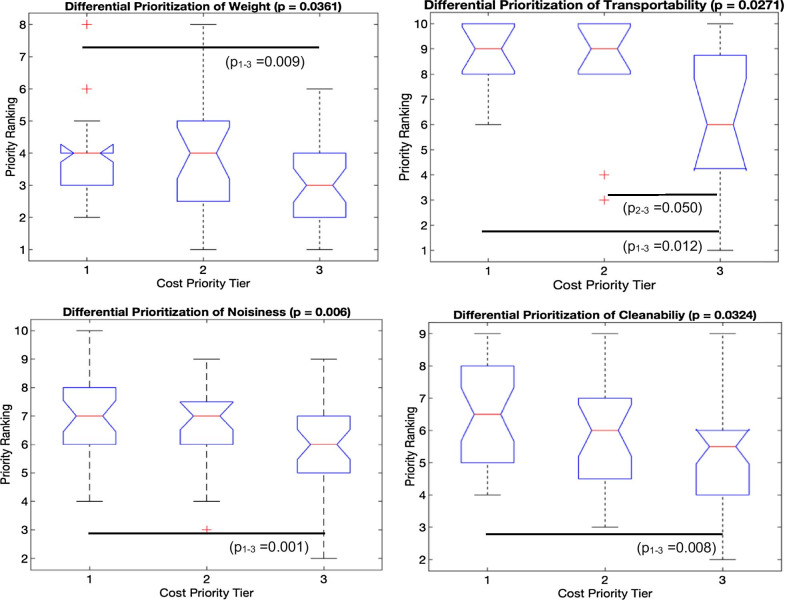
Significantly different user priorities (Pr-GC) across cost-sensitivity tiers. Significance determined by Kruskal Wallis tests across all 3 tiers. Pair-wise significant differences from post-hoc Mann-Whitney U test denoted by horizontal bars. Red lines denote medians, boxes denote inter-quartile ranges, tick marks absolute ranges, with outliers noted as red crosses. *NB:* Higher priorities denoted by *lower* priority rankings

To visualize differences in user priorities as a function of sensitivity to cost, Fig. 4 depicts all users in the PC dimensions 1–3, segmented into 3 tiers based on their cost priority ranking: high (1–3), medium (4–6), and low (7–10). To conceptualize how a given user’s position in PC-space represents their design priorities, it is important to note that for a given PC, *higher* values of the PC denote *higher* values (i.e. *lower prioritization*) of positively weighted variables (Table 10) and *lower* values (i.e.* higher prioritization*) of negatively weighted variables. Apart from the clear distinction between cost sensitivity clusters in the Pr-GC-PC3 dimension (Fig. 4a) due to the dominant weighting of cost priority in this PC, there are no visibly discernible differences in overall distribution of user priorities as a function of cost sensitivity at the principal component level. Nevertheless, Kruskal–Wallis tests for inter-cost-tier differences in individual priorities revealed significant differences for (p < 0.05) for the device characteristics of weight, noise, cleanability, and transportability, as visualized by the box plots in Fig. 5. There were no such significant cost-based differences in Pr-Fn or Pr-AA. Regarding the role of income in determining cost sensitivity, correlation analysis found no significant correlation between income tier and prioritization of cost (rho = 0.085; p = 0.44), whereas cost prioritization exhibited a weak yet significant correlation with the degree of financial assistance, based on prosthesis payer type (rho = 0.392; p = 0.0003).

**Fig. 6:**
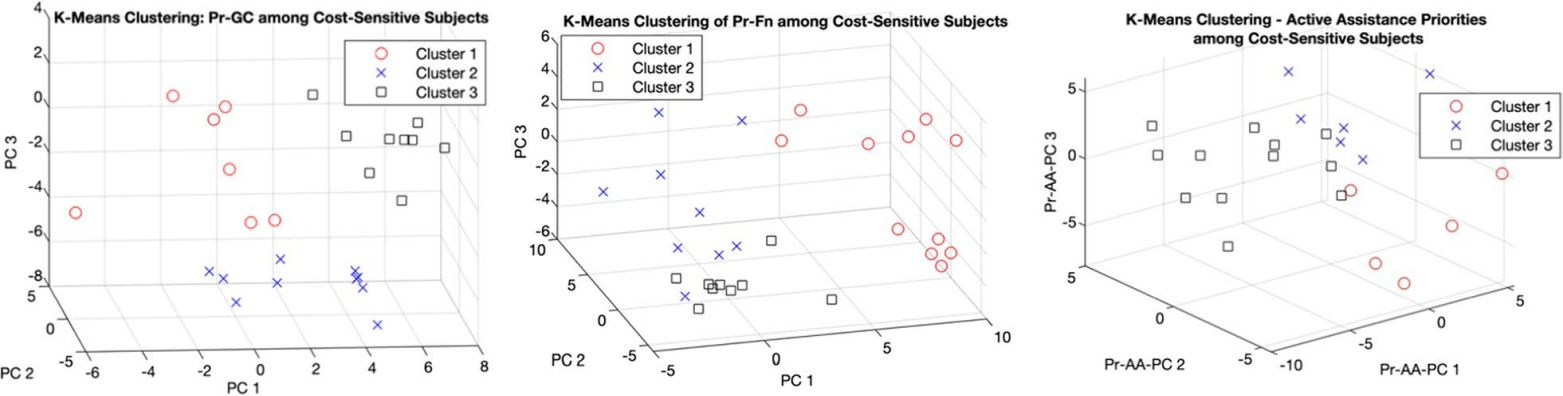
Sub-segmentation of cost-sensitive subjects via K-means clustering (visualized in PC space)

To further investigate potential groupings among the most cost-sensitive subjects, targeted K Means clustering of users in the most cost-sensitive tier yielded three visually distinct, minimally overlapping user clusters in Pr-GC, Pr-Fn, and Pr-AA categories, as displayed in Fig. 6. The relative locations and separation of these clusters in PC-3 space can be understood in terms of the dominant variable weightings (both positive and negative) in each cluster, as described above with respect to Fig. 4. For example, among cost-sensitive users in the Pr-GC category, Cluster 1 is distinguished from Cluster 3 primarily by lower values in the Pr-GC-PC1 dimension (Fig. 6a), which represents a trend towards higher prioritization (lower priority number) for aesthetic aspects and noisiness, in exchange for lower battery performance and transportability. Meanwhile, Cluster 1 in Pr-Fn is characterized by higher ratings in Pr-Fn-PC1, representing a tendency towards higher valuation for gait on inclines and declines, with less emphasis on speed of functioning, stair descent, and lifestyle-related functionality.

## Discussion

The current study data provide a transverse characterization of transfemoral prosthesis (TFP) users, their experience and satisfaction with their current prostheses, and their priorities for an ideal prosthesis, from which we can draw numerous insights pertinent to the user-centered design of technologically-advanced TFPs.

### User characteristics

The present study cohort presented a range of ages, limb loss etiologies, occupations, employment statuses, and income levels (Tables 2 and 3), reflecting the diversity of the TFA population. A salient feature of the subject sample was the predominance (78.9%) of traumatic causes of amputation, differing markedly from the estimated 16.4% of TFA among total lower-limb amputations [4]. In addition to potential differences in the prevalence of transfemoral vs. below-knee cases among traumatic vs. non-traumatic amputations, this predominance of traumatic and male amputees likely represents a recruitment bias at the primary survey administration site, the INAIL Prosthetic Center—a rehabilitation center for patients with work-related disabilities—an effect reported in previous studies with a predominance of TFAs [28]. Moreover, the significantly greater portion of traumatic amputations among MPK relative to NMPK users is likely indicative of differential prosthesis selection based on overall patient health and activity level (presumably higher among traumatic cases due to younger age and lower prevalence of systemic co-morbidities).

Given the difference in amputation etiology distribution between the MPK and NMPK groups, the observed differences in age at amputation and time since amputation may be partially attributable to the contrasting clinical circumstances and pre-amputation lifestyles commonly associated with traumatic vs. non-traumatic (typically *dysvascular*) amputations. Whereas traumatic amputations are commonly associated with accidents during rigorous physical activity, non-traumatic amputations are overwhelmingly the result of vascular disease occurring secondary to many years of chronic metabolic diseases such as type II diabetes [41], which is strongly associated with sedentary lifestyles.

### User experience & satisfaction

In aggregate, our survey data indicated consistently that subjects with MPKs use their prostheses significantly more frequently (Table 5: daily and weekly), experience a significantly greater sense of autonomy (Fig. 1, Table 6), and are overall more satisfied with their devices (Fig. 2, Table 8) than those with NMPK-TFPs. Specific notable findings and implications are discussed below, by category.

Achieving independence in ADLs and a corresponding sense of personal autonomy is a primary clinical objective of prosthesis use, thus requiring routine, independent prosthesis usage in a range of activities and environments [23, 42–44]. To this effect, our study sample shows a significant positive correlation of weak-to-moderate strength (rho = 0.277, p = 0.0001, Table 9) between greater daily prosthesis utilization and greater autonomy among all participants. Moreover, the significantly greater frequency of prosthesis usage relative to NMPKs across home, work, and recreational environments (Table 5) implies both that MPKs may more effectively support the functional autonomy of TFA users over a wide range of activities, and that they may promote a greater overall level of activity. Notably, this effect was strongest for the home environment (f = 0.783), followed by recreational contexts (f = 0.717), followed by work (f = 0.655)—thus implying the greatest differential benefit of MPKs in unstructured personal environments.

Regarding the distribution and differences in reported autonomy between groups, NMPK users exhibited greater variance (IQR) relative to MPK users, both within individual ADL and across all ADLs (Fig. 1; Table 6). In addition to suggesting potentially greater diversity among NMPK users in terms of overall health and function, this trend towards significantly greater autonomy and satisfaction with prosthesis functionality (with mild-to-moderate effect sizes) among MPK users also suggests the possibility that MPK use could facilitate convergence towards positive functional outcomes across a wide range of baseline user health characteristics. Indeed, the persistence of greater autonomy among MPK vs. NMPK users *within* both the demographically self-similar traumatic and non-traumatic sub-groups (Table 6) supports the latter hypothesis, that the prosthesis could play a significant role in facilitating autonomy in the context of a proactive, personalized clinical rehabilitation program. In particular, the more pronounced differences in autonomy (i.e. stronger effect sizes) between MPK and NMPK users with non-traumatic amputations suggests that patients with lower baseline health and/or functional capabilities may draw additional clinical benefit from the use of technologically advanced TFPs. By contrast, the positive differences in autonomy associated with MPK use among traumatic amputees were generally subtler (often evident in the variance rather than the median) and more task-specific, with the greatest differences observed for the more challenging tasks of stair descent, sit-stand transitions, and ramp walking, in addition to housework. To assess the relative contribution of the prosthesis type to these differences in outcomes in light of inherent differences in overall health and physical ability between traumatic and non-traumatic TFA, it would be worthwhile to further investigate the potential improvements in autonomy that may be achieved by assigning MPKs to users who are typically provided NMPKs in the current clinical paradigm. Such a study could in turn support the refinement of clinical care guidelines on prosthesis selection in line with ever-advancing technological capabilities, so as to achieve better functional outcomes for more patients.

As a concrete clinical outcome complementary to subjective autonomy, the higher reported incidence of falls among NMPK users in the present study did not reach significance (Table 7), which must likewise be considered within a complex interplay of technical and clinical factors. In particular, fall risk among lower-limb amputees has been previously related to various biomechanical factors, including gait asymmetry, muscle weakness, and other neuro-musculoskeletal limitations [45, 46], as well as to environmental factors such as irregular terrain, stairs, and slopes [47, 48]. The prosthesis plays a crucial role in safely negotiating these ‘challenge scenarios,’ with previous studies showing that the use of MPK prostheses can improve motor functions and reduce falls in amputees with lower mobility grades [49, 50], in addition to promoting greater overall movement control, dynamic stability, and functional mobility [3, 11, 51]. As a consequence, users of MPK on the whole may become more active in more challenging environments, thus diminishing the net effect of the prosthesis their incidence of falls. Nevertheless, incidence of falls and functional performance parameters such as walking speed on various surfaces and stair descent ability have all been linked with overall amputee satisfaction, wellbeing, and quality of life [11, 49, 50, 52].

The current study findings thus reinforce an integrated clinical picture in which technologically advanced TFPs can be powerful tools in promoting user mobility and autonomy, but one that must be employed as part of a comprehensive rehabilitation paradigm that emphasizes functional training in addition to proactive psychological support. Further evidence for such an integrated approach is found in this study’s observation that users of MPKs, via their common attribution of falls to personal and attentional rather than environmental or technical factors, exhibited a higher degree of control and ownership of their prostheses, thus highlighting the symbiotic relationship between psychological factors and functional outcomes. Based on the preponderance of past and present evidence, it is reasonable to infer that more advanced TFPs can be more effective than traditional TFPs at realizing user potential that depends simultaneously on various health and dispositional factors, and that thoughtful prosthesis selection and configuration based on individual user needs will thus remain necessary to maximizing the benefit of such devices.

This study’s principal finding that strong majorities of TFP users in both groups regarded their prostheses either as useful tools for achieving personal autonomy or as extensions of their bodies is a positive indicator of successful functional rehabilitation and prosthesis acceptance in the study population. Moreover, the increased sense of anatomical *ownership* (“part of me”) expressed by MPK users suggests that both naturalistic prosthesis function and the experience of *using* it in a synergistic manner may contribute strongly to the sense of body schema integration and corresponding acceptance. This implication echoes previous studies that have identified human interaction as a strong factor in improving the subjective sense of control of the artificial leg, thus enhancing the performance and management of ADLs [30, 53].

This study’s observation of significantly higher prosthesis satisfaction with a moderate effect size among MPK relative to NMPK users in all four categories of functionality, comfort, aesthetics, and general characteristics (Table 8) aligns with previous research that has found overall satisfaction to be influenced by several aspects of the prosthesis, including functionality, cosmetics, and usability [31, 44]. While it may be expected that traumatic amputees (91% of the MPK group, vs. just 60% of NMPKs) tend to be more functionally capable than those with dysvascular amputations by virtue of better overall health, it may likewise be true that less healthy and less active individuals have lower mobility *demands* and/or *expectations*, thus making the net effect of prosthesis type on *satisfaction* unclear a priori. This complexity implies both that TFPs should be designed with more than bare-minimum functional autonomy requirements in mind (especially with respect to gait), and also that user satisfaction can be positively influenced by psychological counseling featuring proactive management of expectations and attitudes, regardless of prosthesis type. In sum, the weak-yet-significant positive correlations (Table 9) between prosthesis usage, autonomy, and all aspects of prosthesis satisfaction (functional; practical; aesthetic; comfort), with the strongest relationships between autonomy and aesthetic aspects, mobility-related functionality and general characteristics, further reinforces that higher TFP performance is just one of many factors that significantly influence functional outcomes, overall user satisfaction, and wellness.

### Priorities for an ideal transfemoral prosthesis (TFP)

Given the critical role of device design in prosthesis usability and user satisfaction, the evaluation of user priorities represents an indispensable foundation of the TFP design process. Design priorities reflect the user’s values, lifestyle, and goals for prosthesis use, which have been found to vary significantly by prosthesis type and user age in upper limb prostheses [44]. While the median user priorities in the current study likewise varied between MPK and NMPK users (Fig. 3), the magnitude and significance of these differences were highly task-specific and appear secondary to the high variation in priorities *between individuals*, reflecting the diversity in subjects’ demographic/clinical characteristics (Tables 2 and 3), autonomy (Fig. 1; Table 6), satisfaction with functional ability (Table 8) evidenced by other survey sections. Despite the high variance in user priorities for specific features, common high-level groupings of priorities across all subjects remain pertinent and informative to TFP design.

*Priorities for functional mobility (Pr-Fn)* The prevailing functional priority of general stability across all TFP users agrees with previous findings among MPK users [50]. Here, NMPK users were more consistent in ranking stability first (mRO 1(1.25) NMPK vs. 2(3) MPK, Fig. 3), whereas MPK users as a whole expressed comparably strong preferences for stability and lifestyle adaptability (mRO 2.5(3.75)). Given that MPK users reported significantly greater overall autonomy and satisfaction with functional mobility than NMPK users, this subtle difference suggests that design priorities are influenced both by user lifestyle (actual and desired), and also by user perceptions about the *limitations* of their current prostheses relative to their expectations of device capability. Moreover, we note that the highest-ranked functions were those applicable to a range of situations (overall stability, lifestyle-related functionality, adaptability of walking velocity), with more specific tasks of gait on uneven terrain, stair ascent, and ramp walking (up and down) falling in the second tier. Based on our analysis of free responses, “lifestyle functionality” was interpreted by subjects in a variety of ways (some more task-specific than others), thus rendering this priority interpretable in aggregate as a measure of the need for TFP versatility and adaptability to different tasks and environments.

Notably, “work-related functionality” represents an exception to the trend towards favoring versatility, ranking as a moderate-to-lower priority for both groups. The distinction in preference for lifestyle over work-related functionality is difficult to parse, given that potential differences in functional demands between lifestyle and work environments are not easily generalizable, nor discernible from survey data. What may be inferred regardless is that TFP users consider the ability to maintain their desired personal lifestyle a more important determinant of their satisfaction than their vocational ability.

*Priorities for Active Assistance (Pr-AA)* Overall, TFP user priorities for AA agree well with those for functional mobility, with the top functional priority of overall stability corresponding to the preference for AA during moments of instability. Likewise, the whole-sample trend towards prioritization of ascent vs. descent functions versus their descending analogs in Pr-Fn (Fig. 3) corresponds to the preference for AA in those tasks. This effect is reflected as well in the finding that the most significant differences in Pr-AA between MPK and NMPK users were with respect to stair ascent and incline gait.

The prioritization of different locomotion velocities provides a more nuanced picture: while the high prioritization of adaptability to walking velocity corresponds functionally to the preference for active assistance during fast versus natural versus slow speed walking, the *highest speed* form of locomotion—running—was among the *least* prioritized functionalities by both groups. This finding indicates that high speed walking differs significantly from running in terms of its personal value to users—perhaps owing to a difference in social utility. Given the unique biomechanical demands of running, the elimination of this function as a TFP design requirement would enable a valuable simplification in TFP design.

Regarding the future design and development of advanced TFPs, we note that for both gait speed and ascending vs. descending functions, user priorities for AA reflected the biomechanical demands of the highest-priority mobility functions, with preference to tasks demanding greater positive power output. By contrast, controlled descent and lower-speed gait are more easily achieved via the modulated resistance achievable by current MPKs. This high-level correspondence between priorities for AA and for overall prosthesis functionality suggests that these categories may be strategically merged into a single class of design requirements. In line with user-centered design recommendations from the fields of both lower-limb prosthesis design [30] and brain-computer-interface-based assistive technology [54], such a design process should focus first on defining user priorities regarding the desired tasks and activities to be performed with the prosthesis, based on user input. Specific technical requirements such as active knee power should then be defined based on the biomechanical and ergonomic demands of those tasks, so as to enable users to perform their highest-priority activities in a safe, effective, and efficient manner.

*Priorities for General and Socket Characteristics (Pr-GC; Pr-S)* Current MPK and NMPK users expressed very similar priorities regarding both general device characteristics and socket design. Primary between-group differences in Pr-GC tended to concern more technical, higher-performance TFP traits such as battery life and water resistance (Fig. 3), which likely reflects a difference in *applicability* of various traits to the user’s TFP rather than a fundamental difference in priority. This finding is congruent with previous findings that user priorities vary markedly based on type of prosthesis [31, 44]. Similarly, the only pronounced difference in Pr-S was the elevated preference for active-cooling by MPKs users. This difference is likely attributable to the higher prosthesis usage in this group (Table 5), which suggests higher overall activity levels among MPK users that would naturally result in more frequent sweating and residuum volume changes. The consistency of socket-related priorities across groups supports the modular design of high-performance sockets that are compatible with a wide range of prosthetic knee types, suitable to a range of activity levels, based on user lifestyle.

### User segmentation via clustering of design priorities

The principal component analysis (Table 10) and subsequent K-means clustering of users by design priorities (Fig. 6) present a means of understanding and navigating the individual variation in user priorities. First, the possibility for substantial dimensionality reduction (PVC parameter, Table 10) and the functionally coherent groupings of variable weights within the primary PCs (Table 10, Additional file 1: S1–S3) enable the interpretation of the PCs as representing different ‘functional primitives’ in user priorities, analogous to the concept of dynamic movement primitives [55]. For instance, while Pr-AA-PC1’s primary positive weighting of sit-to-stand and stand-to-sit transitions may be interpreted together as an aggregate index of sit-stand transition priority, Pr-AA-PC2 (positively weighted for gait on level ground and at normal speed) represents normal locomotion, and Pr-AA-PC3 (weighted for incline gait and stair ascent) represents ascending forms of locomotion.

This functional coherence among the key dimensions of user variability carries positive implications for the design and clinical personalization of prostheses, by enabling the interpretation of PCs as meaningful summary parameters representing distinct, functionally related subsets of tasks or device features. Taken together with the high compressibility of survey data, this functional coherence facilitates the creation of a consolidated set of functionally integrated performance measures. If evaluated in a sufficiently standardized fashion (e.g. via a clinically validated survey based on the one used in this study), these metrics could potentially serve as both ‘benchmark’ measures to inform the optimal design of advanced TFPs, and as a tools for clinical care personalization. By evaluating TFAs along the primary dimensions of variation in personal priorities, such a survey could be a powerful tool in the selection and personalization of the available prosthesis that best fulfills the individual’s needs, thus offering dramatic improvement over the “K-Levels” used currently for this purpose, which are limited by their lack of both objectivity and standard assessment criteria [56].

While useful for visualizing and conceptualizing the similarities, differences, and variance in user priorities between different user subsets, this method of generalizing PC interpretation presents a number of limitations. First, the largely contiguous (non-separated) nature of some clusters highlights that user priorities fall along a continuum, making it difficult to draw distinct design boundaries corresponding to discrete performance tradeoffs. Second, each PC encodes information from *all* variables, so the functional interpretation of PCs Table 10 entails some simplifications, some of which are cleaner and more representative than others. Finally, the sub-clustering of cost sensitive users might be more powerful if based on a set of PCs derived specifically for the cost-sensitive segment of the TFP population.

*Cost sensitivity sub-analysis*

While the segmentation of TFP users by their prioritization of cost did not result in a clean separation of users in the overall priority space summarized by the primary PCs (Fig. 5), the detection of significant across-segment differences in the individual priorities of weight, transportability, noisiness, and cleanability (Fig. 5) provides some useful design guidance. Specifically, the most cost-sensitive users rated each of these other design characteristics as significantly less important (higher ranking number) relative to the least cost-sensitive users, while differences between highly and marginally cost sensitive users appeared marginal and insignificant. Nevertheless, these differences in priorities across cost sensitivity tiers suggest the relaxation of these specific design requirements in lower-cost TFPs. Less stringent weight requirements provide greater flexibility in the choice of mechanisms and materials, while prosthesis noise, cleanability, and transportability represent more superficial characteristics of overall lower priority that can be compromised without significantly affect device performance.

The targeted sub-clustering of users within the most cost-sensitive user segment (Fig. 6) provides a further means for exploring possible tradeoffs in the features and functions of a low-cost TFP. By embedding higher-dimensional sets of priorities in a functionally relevant manner, the PCs enable the interpretation of the user segmentations of the most cost-sensitive users in the Pr-GC, Pr-Fn, and Pr-Fn categories (Fig. 6). Overall, the emergence of visually distinct, minimally overlapping clusters in each 3-PC dimensional space suggests the possibility to optimize different prosthesis versions or configurations for different user sub-segments. For example, Cluster 1 in Pr-Fn space is distinguished by higher Pr-Fn-PC1 values, corresponding to a higher prioritization of gait on sloped surfaces, while caring less about the prosthesis’ speed of functioning, ability to descend stairs, and lifestyle-related functionality (versatility). Thus, a lower cost TFP targeted for this user segment may aim to satisfy challenging design tradeoffs imposed by the cost constraint by favoring a device design specialized for incline gait, with less versatility and slower processing/reactions times. Such targeted user-centered insights may be used to guide the design process in a manner that’s non-obvious from the traditional design perspective of trying to maximize prosthesis performance over the general highest-priority functions for the overall user population. Such targeted tradeoffs can be especially impactful towards lowering prosthesis cost for the most cost-sensitive prosthesis users and payers.

Finally, regarding the influence of demographic characteristics on cost cost-sensitivity, the lack of significant correlation between income and cost priority is explained by the significant correlation between the cost priority and subjects’ level of financial support (insurance) in purchasing the prosthesis. This finding simultaneously emphasizes the important role of health insurance in prosthesis selection, both via direct influence on user priorities and via potential differences in reimbursement of different devices, which can vary significantly by region. Furthermore, we note that the individual user’s sensitivity to cost may not fully capture the net cost constraint imposed by healthcare systems and medical payers.

### Application to user-centered design of TFPs

Following from the above discussion, this study’s findings may be synthesized into the following recommendations for the user-centered design of an ideal TFP:

- The safety and reliability of the TFP across a wide range of ADLs are fundamental design priorities for a strong majority of TFA users, preserved across all prosthesis types and other design priorities.
- Individual user needs and priorities vary significantly based on clinical characteristics, personal attitudes, and lifestyle, thus demanding modularity and/or customizability of various prosthesis components and characteristics, such as the dimensions, socket fit, and cosmetics. Indeed, a comprehensive design process should account for the interactions between these components and their net effect on both functionality and satisfaction, as recent studies have found components such as the socket to play a significant role in overall user function with the prosthesis—including gait speed and risk of falls [57].
- A high prioritization of “lifestyle-related functionality” may be interpreted as a desire for functional versatility and adaptability to different activities and unstructured real-world environments. This presents a host of complex design challenges that calls for a combination of modular TFP designs and intelligent adaptive control strategies, enabling device personalization based individual user capabilities and preferences, with the understanding that performance tradeoffs are inevitable. The exploration of such tradeoffs—including the dynamic, interdependent relationship between prosthesis hardware and control—has been well exemplified and further enabled on a larger scale by the recent development of open-source bionic leg by Azocar, Hargrove, Rouse, and colleagues [58].
- The prospective value of active (i.e. positive power) assistance to TFP users varies by task, with assistance desired preferentially during moments of instability, stair/incline *ascent*, and higher-velocity walking.
- Based on the leading prioritization of shape/volume adaptability among both MPK and NMPK users, the ideal TFP socket design should enable routine modulation of shape and/or volume to accommodate changes in residual limb volume and tissue properties, thus improving comfort and minimizing skin problems. In conjunction, socket designs that achieve smart regulation of temperature and humidity (second priority, Fig. 3) are highly desirable.
- The optimization of prosthesis weight relative to its function (e.g. achieving a high torque-weight ratio [3]) remains an important design objective for many users. However, this priority varies in a significantly inverse fashion with prioritization of cost, thus offering a design requirement that may be loosened in the case of lower-cost, high-preforming TFPs.
- The prioritization of cost as a factor for prosthesis selection is highly variable among TFAs, depending more strongly on insurance reimbursement than on income per se. While user priorities regarding prosthesis functionality and general prosthesis characteristics do not vary significantly according to cost sensitivity, the most cost-sensitive users are distinguished from the least cost-sensitive users by their lower prioritizations of prosthesis weight, transportability, noisiness, and cleanability. Given the limiting role of cost with regard to prosthesis functionality, lower-cost TFPs intended for cost-sensitive users should thus focus on overall stability, reliability, and comfort as characteristics with the greatest impact on quality of life, while maintaining secondary priorities within a safe, functional, and user-acceptable range.
- For the efficient diversification of low-cost prosthesis designs, we recommend that design alternatives be developed in alignment with the functional groupings of user priorities represented by the dominant variable weights in the leading principal components identified in this study.

To fulfill these requirements in the development of future TFP systems, they should be used by TFP researchers and developers as design inputs to a rigorous user-centered design framework such as that proposed by Beckerle and colleagues [30], which posits a systematic process for merging human and technical factors in the design of advanced lower limb prostheses, with particular attention to TFP. Such a process should further explore the functional improvements achieved via advances in TFP hardware relative to those achieved via improved sensing, intention detection, and control strategies—both of which are fundamental to the performance of robotic MPKs [58]. The relationships between key TFP design parameters in all of these domains (hardware, sensing, and control), user preferences, and functional/clinical outcomes should be further explored, as Clites, Rouse, and colleagues have recently investigated for the parameter of prosthetic ankle stiffness [59]. Finally, to make such research relevant to ongoing advancements in neuroprosthesis technology, the relative benefits of various TFP design features and performance settings should also be investigated in the context of advanced neuromuscular integration approaches such as targeted muscle reinnervation and intramuscular electrodes for both prosthesis control and sensory feedback, including the case of osseointegrated prostheses as well [60].

In addition to the above recommendations regarding TFP design characteristics, the present study reveals several valuable insights regarding the human-centered design *process*. First, the survey’s ranking of user design prioritizes without any corresponding measures of relative priority *weighting* favored the delineation between TFP features of similar priority, with the tradeoff of reducing the power to evaluate the absolute *importance* of specific design features. By contrast, non-static ranking schemes such as Best–Worst or MaxDiff scaling [61, 62] may enable more meaningful prioritization among targeted subsets of features. Second, survey questions regarding subjects’ functional capabilities were phrased in terms of subjective *satisfaction* and autonomy, making them imprecise as indicators of absolute functionality. Though this perspective is suitable for a user-centered design process that holds user satisfaction and wellbeing as its ultimate objectives, subsequent TFP research and development efforts should further investigate the relationship between specific design characteristics, objective functional performance, usability, and user satisfaction. Finally, future studies should evaluate user priorities in a manner that more directly aligns with the actual technical tradeoffs that constrain TFP design, such as the fundamental tradeoffs between weight, power, and functional versatility.

## Study limitations

While supportive of the current clinical understanding that more advanced TFPs promote higher levels of user mobility, function, and overall health, this study’s central findings of higher overall functional autonomy and satisfaction among MPK relative to NMPK users must be interpreted within the context of two main study limitations. Most notably, owing to the survey’s limited characterization of numerous health-related co-factors such as participants’ overall health, lifestyle (via pre- and post-amputation occupation), and objective functional capacity (as quantified by standard clinical measures such as the K-level or functional assessment scales), the study was not able to conduct a thoroughly controlled test of the hypothesis that MPKs confer independent improvements in outcomes relative to NMPKs, which was beyond the scope of objectives for which the survey was designed. Indeed, perceived autonomy, satisfaction, and user priorities may all be expected to depend significantly on the relationship between the individual’s pre- and post-amputation occupation and lifestyle, which strongly influence personal identity. Satisfaction and priorities may further be influenced by the individual’s conception of what level of functional recovery is possible in the context of the current clinical and technological state of the art. Irrespective of these survey-specific constraints, it remains likely that the effects of MPK-TFPs on clinical and personal outcomes cannot be thoroughly isolated from general health and human factors even by controlling for primary indicators of such factors, due to the number and the limited quantifiability of personal factors that influence prosthesis selection.

Second, the aforementioned recruitment bias towards traumatic TFAs limits the generalizability of full-sample analyses to the general TFA population. Moreover, the primary study site (INAIL Centro Protesi) is the leading orthopedic rehabilitation center in Italy, where the intensive, integrative standard of care creates a patient population likely more functionally advanced and engaged with their prostheses than the global TFA population. Indeed, the time course, quality, and user engagement in prosthetic rehabilitation is known to strongly influence clinical and functional outcomes irrespective of prosthesis type, thus influencing a range of psychosocial outcomes as well. This center’s standard care practice of prescribing MPKs *following* outfitting and initial rehabilitation with an NMPK-TFP may introduce an additional “healthy user bias” [63] among MPK users. Specifically, patients with a lower overall clinical health status and/or lower mobility according to Medicare Functional Classification Levels (MFCL) are typically issued and conduct initial functional rehabilitation with a simpler, lower-weight mechanical (NMPK) prosthesis—characteristics understood to improve ease of use and the user’s feeling of safety in using the device. This type of device is also characterized by a lower price, which is an important factor in the Italian national health system, as it allows the patient to try the prosthesis with full reimbursement, making it accessible to all without sacrifice. By contrast, while healthier users are presumably able to achieve the best functional outcomes from advanced TFPs, it remains possible that such devices could have the greatest *marginal benefit* for users with lower baseline abilities, as suggested by autonomy data among non-traumatic amputees (Table 6). Nonetheless, this study’s heavy representation of traumatic amputees and MPK users is well-suited to the study’s objective of evaluating user needs and priorities for the development of an advanced, actively powered TFP.

## Conclusions

This study provides an extensive new characterization of transfemoral amputees (TFAs), encompassing their demographic and clinical characteristics, psychosocial attitudes and lifestyles, functionality and satisfaction with their current prostheses, and user needs and priorities for an ideal transfemoral prosthesis (TFP). Overall, users of TFPs with microprocessor-controlled knee units (MPKs) reported higher levels of activity, prosthesis use, and functional ability compared to those with NMPKs, corresponding to higher satisfaction and greater functional autonomy. Significantly, While likely reflective of differences between MPK and NMPK users in terms of age and amputation etiology (and thus, overall health), these results are consistent with the hypothesis that more advanced, actively controlled TFPs positively influence not only the safety and functional mobility of TFAs, but their overall sense of prosthesis acceptance and ownership, personal autonomy, and overall health and wellbeing. Moreover, this study’s finding that significant differences in ADL-related autonomy for MPK vs. NMPK users were higher in magnitude among non-traumatic relative to traumatic amputees suggests that TFP users with a lower overall health status may in fact obtain greater marginal benefit from the use of advanced TFPs, and that the current standard-of-care guideline of prescribing MPK prostheses selectively to more capable users should thus be more carefully evaluated. Based on past and present findings, it may be reasonably generalized that advanced TFP functionality, in conjunction with a variety of underlying clinical and personal factors, plays a significant role in *enabling*, *maintaining*, and/or *reinforcing* healthy mobility and lifestyle among TFAs.

Finally, a set of user-based design principles are synthesized based on survey data analysis. Future investigations should continue to develop, validate, and standardize measures of the functional abilities and personal priorities of TFAs, based on this study’s survey and findings. In this way, this study may serve as a foundation to build a clinical evaluation tool to optimally select and configure TFPs to fulfill the needs of individual users.

## Supplementary Information

**Additional file 1: Table S1.** Principal Component Loadings—User priorities for general characteristics of an ideal TFP (Pr-GC). **Table S2.** Principal Component Loadings—User priorities for functionality of an ideal TFP (Pr-Fn). **Table S3.** Principal Component Loadings—User priorities for active assistance in an ideal TFP (Pr-AA).

## Data Availability

The dataset supporting the conclusions of this article is not publicly available due to subject privacy considerations (per ethics committee approval) but may be obtained from the corresponding author upon reasonable request.

## Abbreviations

AA: Active Assistance
ADL: Activities of Daily Living
IQR: Inter-Quartile Range
MPK: MicroProcessor-controlled Knee unit
mRO: Median rank order
NMPK: Non-MicroProcessor-controlled Knee
PC(A): Principal component (analysis)
Pr-AA: Priorities for activities during which to receive Active Assistance from an ideal prosthesis
Pr-GC: Priorities for General Characteristics of an ideal prosthesis
Pr-Fn: Priorities for the mobility-related Functionality of an ideal prosthesis
Pr-S: Priorities regarding the Socket of an ideal prosthesis
SACH: Solid Ankle, Cushioned Heel
TFA: Trans-Femoral Amputee
TFP: Trans-Femoral Prosthesis
