# Supplementary material for: Survey of transfemoral amputee experience and priorities for the user-centered design of powered robotic transfemoral prostheses
Source: J Neuroeng Rehabil. 2021 Dec 4;18:168. doi: 10.1186/s12984-021-00944-x (PMC8643009; doi:10.1186/s12984-021-00944-x)
Supplement: Supplementary file 1 — Additional file 1: Table S1. Principal Component Loadings—User priorities for general characteristics of an ideal TFP (Pr-GC). Table S2. Principal Component Loadings—User priorities for functionality of an ideal TFP (Pr-Fn). Table S3. Principal Component Loadings—User priorities for active assistance in an ideal TFP (Pr-AA). [file 12984_2021_944_MOESM1_ESM.docx]

**Additional Tables**

| Table S.1: Principal Component Loadings – User priorities for general characteristics of an ideal TFP (Pr-GC) | | | | | | |
| --- | --- | --- | --- | --- | --- | --- |
|  | **Principal Component ID** | | | | | |
| **Function** | Pr-GC-PC1 | Pr-GC-PC2 | Pr-GC-PC 3 | Pr-GC-PC4 | Pr-GC-PC5 | Pr-GC-PC6 |
| Cost | -0.030 | -0.262 | **0.674** | 0.110 | 0.302 | -0.088 |
| Aesthetics | **-0.385** | -0.245 | 0.015 | 0.004 | **-0.429** | **-0.487** |
| Comfort | 0.067 | **-0.439** | -0.370 | -0.013 | 0.380 | 0.319 |
| Weight | -0.214 | 0.393 | -0.101 | **0.550** | -0.285 | 0.259 |
| Reliability | 0.110 | **0.446** | -0.168 | **-0.496** | 0.059 | -0.420 |
| Noisiness | **-0.306** | -0.132 | -0.145 | **-0.529** | -0.297 | **0.490** |
| Battery Life | **0.615** | -0.044 | -0.024 | -0.135 | -0.093 | -0.045 |
| Water Resistance | 0.010 | **0.545** | 0.199 | -0.047 | 0.250 | 0.284 |
| Cleanability | -0.215 | 0.028 | **-0.524** | 0.236 | **0.454** | -0.295 |
| Transportability | **0.519** | -0.096 | -0.186 | 0.290 | -0.362 | 0.002 |
| Variance Captured (%) | 20.3 | 17.4 | 14.8 | 11.9 | 9.8 | 9.3 |
| Variance-Cumulative (%) | 20.3 | 37.7 | 52.5 | 64.4 | 74.2 | 83.4 |

| Table S.2: Principal Component Loadings – User priorities for functionality of an ideal TFP (Pr-Fn) | | | | | |
| --- | --- | --- | --- | --- | --- |
|  | **Principal Component ID** | | | | |
| **Function** | Pr-Fn-PC1 | Pr-Fn-PC2 | Pr-Fn-PC3 | Pr-Fn-PC4 | Pr-Fn-PC5* |
| Stability of Support | -0.026 | 0.279 | **0.716** | 0.044 | 0.098 |
| Function re: Work | -0.078 | **-0.490** | 0.051 | **0.723** | -0.195 |
| Function Re: Lifestyle | **0.481** | -0.113 | -0.049 | -0.053 | -0.099 |
| Speed of Function | **0.443** | -0.098 | -0.178 | -0.213 | 0.113 |
| Adaptability to Gait Velocity | -0.195 | -0.337 | 0.225 | -0.273 | **0.670** |
| Uneven Terrain | -0.283 | -0.194 | -0.117 | **-0.483** | **-0.519** |
| Stair Ascent | -0.081 | **0.704** | -0.221 | 0.225 | 0.038 |
| Stair Descent | **0.467** | 0.083 | 0.108 | -0.164 | -0.118 |
| Running | -0.268 | 0.023 | **-0.536** | -0.006 | 0.331 |
| Incline & Decline | -0.389 | 0.074 | 0.194 | -0.207 | -0.296 |
| % Variance Captured | 37.8 | 14.5 | 13.4 | 11.1 | 9.5 |
| % Variance Captured-Cumulative | 37.8 | 52.3 | 65.7 | 76.7 | 86.2 |

| Table S.3: Principal Component Loadings – User priorities for functions during which to receive active assistance from an ideal TFP (Pr-AA) | | | | | | |
| --- | --- | --- | --- | --- | --- | --- |
|  | **Principal Component ID** | | | | | |
| **Function** | Pr-AA-PC1 | Pr-AA-PC 2 | Pr-AA-PC 3 | Pr-AA-PC 4 | Pr-AA-PC 5 | Pr-AA-PC 6 |
| Sit-to-Stand | **0.586** | -0.137 | -0.061 | -0.063 | 0.003 | -0.011 |
| Stand-to-Sit | **0.541** | -0.090 | -0.176 | -0.154 | 0.104 | 0.216 |
| Gait – level ground | -0.070 | **0.489** | -0.071 | -0.114 | **0.546** | -0.239 |
| Gait – level ground at usual speed | -0.110 | **0.550** | -0.129 | 0.307 | -0.021 | -0.128 |
| Gait – somewhat fast | -0.311 | 0.222 | -0.113 | **-0.474** | **-0.536** | 0.396 |
| Incline | -0.095 | -0.053 | **0.581** | 0.344 | 0.175 | **0.519** |
| Decline | -0.367 | -0.356 | -0.212 | 0.062 | **0.434** | 0.278 |
| Stair Ascent | -0.021 | -0.107 | **0.649** | -0.110 | -0.179 | **-0.470** |
| Stair Descent | -0.313 | **-0.430** | -0.122 | -0.339 | 0.153 | -0.337 |
| Moments of instability/  loss of balance | -0.087 | -0.235 | -0.335 | **0.626** | -0.358 | -0.207 |
| % Variance Captured | 22.4 | 18.4 | 16.1 | 11.1 | 9.6 | 7.5 |
| % Variance Captured-Cumulative | 22.4 | 40.9 | 57.0 | 68.1 | 77.7 | 85.2 |
